# Institutional Notes and News

**Published:** 1910-05-21

**Authors:** 


### INSTITUTIONAL NOTES AND NEWS.

**Warrington Infirmary.**

THE Warrington Infirmary has finished its first hundred years of work, having been opened on April 12, 1810, and, judging from its present condition, the new century it has now begun will have very little chance of outliving the hospital. The President, Sir Gilbert Greenhall, marked the occasion by holding last month a reception in the top ward of the new wing, which is now vacant. Our account of the anniversary has been postponed unavoidably, but we are glad to give now what was perhaps its most important feature from a practical point of view. Mr. J. H. Clarke handed over to Mr. Dakin the first instalment of £1,388 17s. 9d. from the Million Penny Fund Committee. He said he felt sure that as each of the four months passed they would be able to hand over a similar cheque to the Hon. Treasurer. The object of the Penny Fund is to raise £4,000 to pay off the debt, and to leave a small surplus for necessary repairs. Many happy returns of the centenary!

**New Wigan Workhouse Infirmary.**

THE new buildings of the Wigan Workhouse Infirmary were opened early this month. They have been built at a cost of £38,000, and consist of four two-storey pavilions, divided equally between the men's and women's wards, a one-storey ward for maternity cases, and provide accommodation for 291 patients. The nursing staff, 18 in number, has a home in a three-storey building, and there is also a block set apart for the medical officer, the superintendent nurse's rooms, and a large kitchen. This has been built in such a way that, when the main buildings are built also, there will be accommodation enough for the future dining-room. A laundry with its necessary plant, day-rooms, bathrooms, and a special bedroom or two to complete the infirmary. The covered corridor system, somewhat similar to that at the Manchester Royal Infirmary, has been adopted. These details of the design show that the Guardians in abandoning the existing workhouse, which has now done duty for 53 years, have decided on an up-to-date infirmary to replace it. We wish every success to the new institution.

**Bristol General Hospital.**

MR. G. S. FRY, the President of the Bristol General Hospital, whose abilities received civic recognition only last year by the bestowal of the honorary Freemanship, will require them all to meet the financial crisis of his hospital at the present time. By January 1910 a heavy deficit was expected, as the previous year had begun with a debt of £4,361, and subscribers have been following with such lagging efforts that a debt on current expenses was fully anticipated of £3,500. A useful, indeed invaluable, legacy or two tided the hospital over the year, and left the hon. treasurer, much better off than he had feared, with a debt of £2,937. The 2,700 in-patients, together with the 39,522 out-patients, whom the hospital treats during the year, cost in food and ministration £14,500. The amount received from subscriptions in the course of the same period is £11,000, leaving the hon. treasurer to solve the difficulty of £3,500 not coming in as best he can. Bristol is a rich city, and needs a stimulus. Each city has its own peculiarities, its own pet weaknesses of heart, which are open to the knowledge of an old citizen. We hope that Mr. Fry will add to the obligations the hospital has been glad to receive from him for about a quarter of a century by using his experience of Bristol to devise a scheme for raising this money. Knowledge and energy are the two factors of success. Knowledge he and the hospital staff must have; it is not to be doubted that any Bristol inhabitant, who remembers the merchant adventurers of an earlier century, can lack energy, we think.

**Bath Royal Mineral Water Hospital.**

THERE is something more than a record of treatment and expenditure to be found in the latest annual report of the Royal Mineral Water Hospital, Bath, as a glance at this week's columns in the *Hospital World* will show. What that record of facts and figures is, however, we give in this place. The number of patients admitted was 1,211, 84 per cent. of whom were "discharged cured or relieved." Though legacies still, unfortunately, are below the average, the hospital's income has increased slightly, while the expenditure was £245 greater than in 1908, chiefly owing to £208 which were spent on mattresses. The fund for endowing the "South Wales Bed" was increased by £129, for which the working classes of Glamorgan and Monmouth must take the chief credit. The hospital owns a farm and estate at Charmey Down, 22 acres of which have been let on lease to the Council, which asked for it for the purpose of extending its small holdings. Colonel Hendley Kirkwood, who moved the adoption of the report, gave the actual figures of income and expenditure as—total income £6,172, total expenditure £5,300. One of the losses by death particularly to be regretted was that of Mr. W. H. Tyas, who had held faithfully for 19 years the office of dispenser.

**The Story of Ilford Hospital.**

THE HOSPITAL of April 30 described the laying of the foundation-stone of the Ilford Emergency Hospital. We are happy to supplement that by a glance at local history, which will show how the hospital came to be started, and who have to be thanked for the institution Ilford has to-day. Dr. Montagu Houghton, as the record which was placed in the foundation-stone, and which was also published in the local paper from the pen of Mr. W. A. Locks, assures us, was the first to entertain the idea about ten years ago. A committee was formed later which discussed, rejected, and finally evolved a scheme which became practical as soon as the estimate of its cost was defined. That estimate and the difficulty of finding money to pay for it would have remained perhaps unsurmounted if an expert on hospital construction had not appeared in the shape of Dr. Richard Greene. From the date of his arrival, the autumn of 1904, matters began to move, and his paper, read in the Town Hall, gave a genuine impetus to the plan of procedure. Enthusiasm, however, is always cumulative in its effects, and the enthusiasm of Dr. Greene was followed by a decision to hold a carnival in aid of the building fund, which decision proved the source if not of financial prosperity, at any rate of solvency and of income. The carnival began with a Hospital Saturday procession, and has developed since into the huge festival which Ilford people now regard as the annual event of each July. The town at large was thus appealed to, and the former Hospital Committee expanded into the committee of the hundred and one, now known as the Board of Governors. Annual subscribers began early to appear, and Mr. Holcombe Ingleby's gift of £1,000 put the success of Ilford Hospital beyond a doubt. The Carnival Committee has handed already over £3,000 to the governors, thanks largely to Mr. Ben Henderson. Difficulties over the site now arose, when Dr. Greene again proved "the organiser of victory," and he remains the expert adviser he showed himself to be in recommending the adoption of Mr. Banks-Martin's drawings. The Newbury Park site, since added to on Dr. Greene's suggestion, costing £1,822, is now paid for, and the Ilford Emergency Hospital is expected to start work this autumn without debt—a business feat of which all who have known the difficulties, and all who have recognised the public spirit in the neighbourhood, may be proud.

